# Complete Genome Sequence of Novel Polerovirus-Associated RNA Infecting Pepper (*Capsicum* spp.) in South Africa

**DOI:** 10.1128/MRA.01215-20

**Published:** 2021-01-07

**Authors:** W. E. W. Schravesande, J. P. van Wijk, A. Verhage

**Affiliations:** a Rijk Zwaan Breeding B.V., De Lier, The Netherlands; DOE Joint Genome Institute

## Abstract

The complete genome sequence of a novel polerovirus-associated RNA infecting pepper in South Africa was determined. The nucleotide sequence identity of 78.3% with closely related species suggested that this associated RNA was novel, and the name pepper vein yellows virus-associated RNA is proposed for this RNA fragment.

## Abstract (precis)

The complete genome sequence of novel polerovirus-associated RNA infecting pepper in South Africa was determined. The nucleotide sequence identity of 78.3% with closely related species suggested that this associated RNA was novel, and the name Pepper Vein Yellows Virus-associated RNA is proposed for this RNA fragment.

## ANNOUNCEMENT

**FIG 1:**
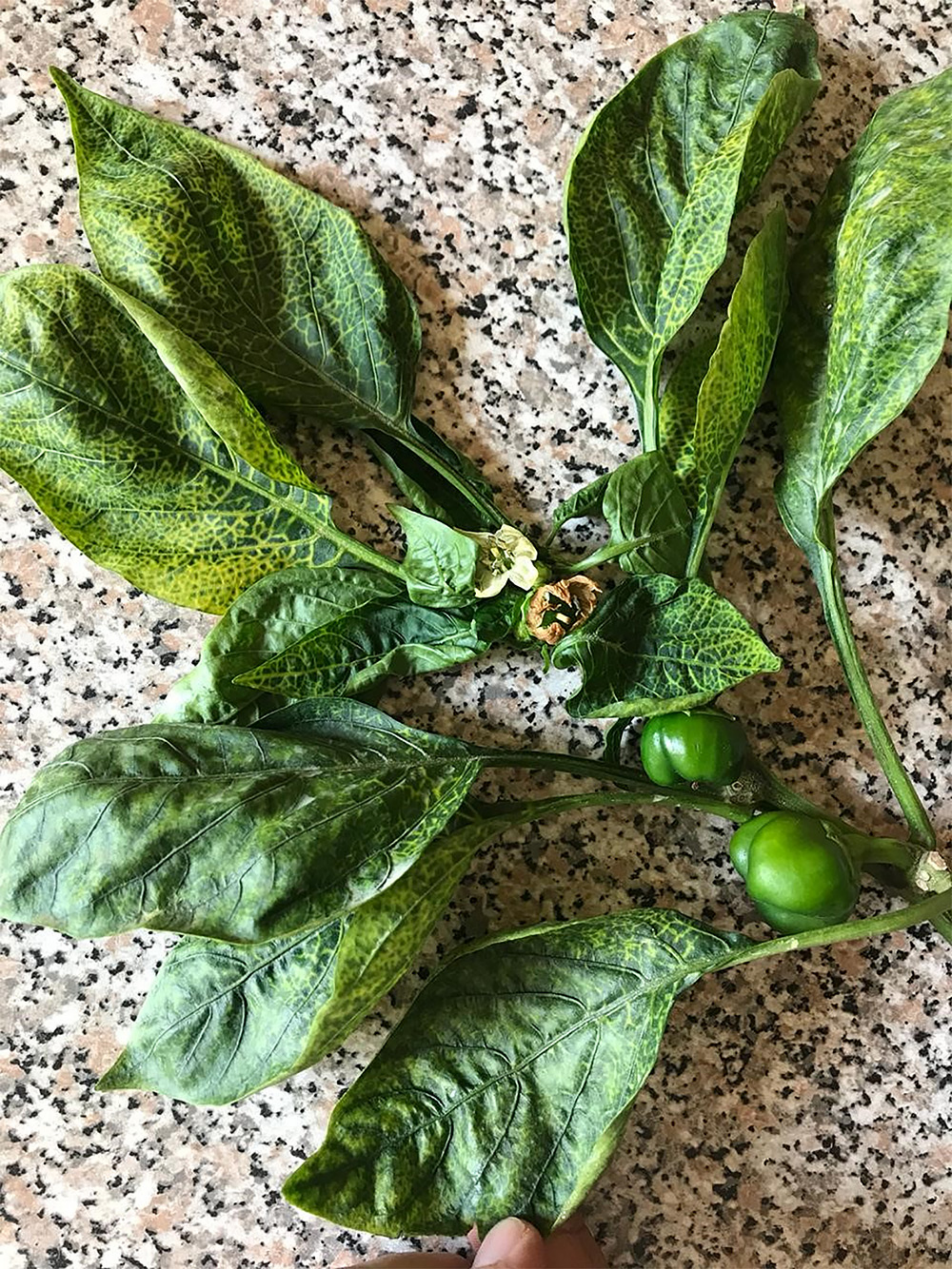
Symptomatic pepper showing yellowing of the leaf veins on the top part of the plant. Additional symptoms include interveinal yellowing and stunting of the growth point (photo by Arno van Heerden).

In February 2020, previously unknown disease symptoms were observed in field-cultivated sweet peppers in Limpopo, South Africa. Symptoms included yellowing of the leaf veins at the top of the plant. As the infection progressed, interveinal yellowing with eventual stunting of the growing tips was observed (Fig. 1).

The observed symptoms led us to screen for begomoviruses, potyviruses, and tospoviruses, with use of Pathogen Immunostrips (Agdia) and enzyme-linked immunosorbent assay (ELISA), which returned a negative result. We therefore started screening for a putative infection with a virus from the genus *Polerovirus*. Symptomatic leaf material was collected, from which total RNA was isolated using the Qiagen RNeasy plant minikit. The RNA was used as the template for double-stranded (ds) cDNA synthesis using the Maxima H Minus ds cDNA kit (Thermo Scientific) using the random hexamers provided by the manufacturer. A sequencing library was constructed following the manufacturers protocol (LSK-109; Oxford Nanopore) and sequenced on the R9.4 MinION flow cells for 72 hours. In short, the double-stranded cDNA was end prepped with use of NEBNext formalin-fixed, paraffin-embedded (FFPE) DNA repair mix (New England BioLabs) and Ultra II end prep mix (New England BioLabs). The DNA was washed with use of AMPure XP beads (Beckman Coulter), and Oxford Nanopore Technologies (ONT) AMX adapters were ligated with use of the NEBNext quick T4 ligase (New England BioLabs). The washing step was repeated, and the library was loaded onto a MinION flow cell. After 72 hours of sequencing, the data were base called with use of Guppy v. 3.6 (Oxford Nanopore Technology) and trimmed/filtered based on length and quality scores (q = 6, minlength = 1,000, headcrop = 20, tailcrop = 20) with use of Porechop v. 0.2.4 (https://github.com/rrwick/porechop) and Nanopolish v. 0.13.2 (https://github.com/jts/nanopolish). The average read length was 1,441 bp, the total number of bases was 7.39 Gbp, and the number of reads was 5.1 million. All tools were run with default parameters unless otherwise specified. The processed reads were assembled using CLC Genomics Workbench v. 12.0 (Qiagen) using the *de novo* assembly option in the long-read module (beta). No polishing steps were executed prior to assembly. Reads originating from pepper were subtracted by mapping the reads to a reference genome (GenBank accession number AYRZ00000000.2). The remaining reads were used for *de novo* assembly. The assembled contigs were used to search the NCBI nucleotide (nt) collection database using BLASTn.

A contig of about 6.1 kb (5,224× mean coverage) showed 94.7% nucleotide similarity to pepper vein yellows virus (GenBank accession number MK931185.1). An additional contig of about 2.9 kb (2,394× mean coverage) was found in the sweet pepper sample, which shares a 78.3% nucleotide identity with the complete sequence of Tobacco bushy top disease-associated RNA (GenBank accession number EF529625.1) (1). Polerovirus-associated RNAs are a class of coat-dependent RNA replicons often found in polerovirus-infected host plants. These associated RNAs replicate autonomously and are encapsidated together with the main genome within the virion, therefore depending on the viral particle for transmission (2). Experiments executed by Sanger et al. (3) and more recently by Campbell et al. (4) revealed that plants coinfected with both beet western yellows virus strain ST9 and ST9-associated RNA displayed increased symptom severity and viral titer, although these observed phenotypes seem to be strain and host specific. Polerovirus-associated RNAs have a size of approximately ∼2.8 to 3 kb and contain two major open reading frames (ORFs). ORF1a codes for a putative protein of ca. 30 kDa. The translational readthrough of the stop codon of ORF1a results in a protein of ca. 89 kDa, which shows characteristics of viral polymerases.

The assembled contig of this new pepper vein yellows virus-associated RNA was polished through Sanger sequencing of additional reverse transcriptase PCR (RT-PCR) amplicons along the genomic RNA. For the amplification of the Sanger sequencing fragments, the following reaction setup was used: 4 µl 5× Phusion high-fidelity (HF) buffer, 0.4 µl 10 mM deoxynucleoside triphosphates (dNTPs), 1 µl 10 mM forward and reverse primer, 0.2 µl Phusion DNA polymerase (New England BioLabs), 50 ng of template cDNA, and nuclease-free water, added to a total volume of 20 µl. The 5′ and 3′ termini of the viral RNA were obtained using rapid amplification of cDNA ends (RACE) technology. The 5′-terminal end was amplified following the manufacturer’s protocol using the 5/3′ RACE kit, 2nd generation (Roche). For the amplification of the 3′ terminus, the molecules were tailed with dATP and TdT. These tailed molecules were used as input material for 3′ RACE using the manufacturers protocol (see Table 1 for RACE and Sanger primers). All Sanger and RACE amplicon sequencing was outsourced. The amplicon sequences were mapped to the prior assembled contig using default settings in CLC Genomic Workbench v. 12.0 (Qiagen).

**TABLE 1:** Primer sequences used for Sanger sequencing and RACE PCR of pepper vein yellows virus-associated RNA

Primer name	Primer sequence (5′–3′)
PeVYV-5′ SP1	CGGAACTCCCTACTAAAG
PeVYV-5′ SP2	CCAGGCAATCCTCTATAC
PeVYV-5′ SP3	GCCATTCCTACACCTAT
PeVYV-3′ SP5	CGTTCACTACTCCTCTAC
PeVYV SANGER FW1	AAACTTAGGAGGAGAGG
PeVYV SANGER RV1	CCAAGAATTCACTTGGG
PeVYV SANGER FW2	GATTCCACTGGCTAAGA
PeVYV SANGER RV2	TCAACCCTCGTTCTATG
PeVYV SANGER FW3	CGAGGGTTGATGAAGTA
PeVYV SANGER RV3	CATCGTTTCCAAGTTCC

The complete genome of the polerovirus-associated RNA with a size of 2,973 nt and a GC content of 50.6% was obtained and further analyzed and annotated by using the Tobacco bushy top disease-associated RNA reference genome under GenBank accession number EF529625.1 as RefSeq in the toolbox Whole Genome Alignment (beta) using default settings in CLC Genomics Workbench v. 12.0 (Qiagen). The genome contains the following two ORFs: an ORF coding for a protein of unknown function of 259 amino acids (aa) and an ORF coding for a putative RNA-dependent RNA polymerase of 786 aa. We propose the name pepper vein yellows virus-associated RNA (PeVYVaRNA) for this RNA fragment.

### Data availability.

The full sequence of PeVYVaRNA isolate PRO54353 can be found in NCBI GenBank under accession number MT321510. Sequencing data are available under SRA accession number SRR12835201.
